# Socioeconomic Inequalities and Multi-Disability among the Population Aged 15–64 Years from 1987 to 2006 in China

**DOI:** 10.3390/ijerph13101033

**Published:** 2016-10-21

**Authors:** Zhenjie Wang, Gong Chen, Chao Guo, Lihua Pang, Xiaoying Zheng

**Affiliations:** 1 Institute of Population Research/WHO Collaborating Center on Reproductive Health and Population Science, Peking University, No.5 Yiheyuan Road, Haidian District, Beijing 100871, China; wangzhenjie1981@aliyun.com (Z.W.); chengong@pku.edu.cn (G.C.); chaoguo@pku.edu.cn (C.G.); pang@pku.edu.cn (L.P.); 2 Laboratory of Neuroscience and Mental Health, Peking University, No.5 Yiheyuan Road, Haidian District, Beijing 100871, China

**Keywords:** multiple disabilities, prevalence, socioeconomic inequalities, China

## Abstract

Socioeconomic inequalities associated with multiple disabilities have not been explored in China. This is the first study to explore changes in multiple disabilities among persons aged 15–64 years in China. Data were derived from the 1987 and 2006 China National Sample Surveys on Disability, which are nationally representative population-based surveys. Both surveys used multistage, stratified, cluster random sampling with probability proportional to size to derive nationally representative samples. We used standard weighting procedures to construct sample weights considering the multistage stratified cluster sampling survey scheme. The impact of socioeconomic inequalities on multiple disabilities was examined by using logistic regression. Higher prevalence rates among rural residents than urban residents were observed. Male was more vulnerable than female in the present study. Minority ethnicity did increase the risk of multiple disabilities, but this association inversed in the logistic regression model. The widening discrepancy between urban and rural areas indicates that the most important priorities of disability prevention in China are to reinforce health promotion and to improve health services in rural communities.

## 1. Introduction

Disability is a common health outcome across the world, in both developed and developing countries [1]. The World Health Organization (WHO) estimates that 650 million people aged 18 and over worldwide live with disabilities [1]. Many people with disabilities across the world do not have equal access to health care, education, and employment, do not receive the disability-related services that they require, and experience exclusion from daily life activities [1]. There are about 92 million people with very significant disabilities in the world, and the presence of multiple disabilities can make the management of health care and rehabilitation services more complicate than single disability can [1]. Although the definition of disabilities was narrower than in other countries in China, it is estimated that approximately 85 million people live with disabilities or another condition that affects their daily lives and social activities [2]. Zheng et al. estimated the number of disabled people was increasing from 52.7 million to 84.6 million, and the weighted prevalence of disabilities also increased from 4.9% to 6.5% accompanying aggressive economic development [3]. Therefore, specific social network, policy and programs are needed to improve the living and health status of those with disabilities, especially for those with multiple disabilities [1].

Socioeconomic inequalities, such as poverty, education, and employment, associated with disability had been confirmed by previous research [1]. Poverty, a crucial measurement of socioeconomic status, was also reported having a positive correlation with disability [1,4,5]. In China, epidemiological studies have conducted specific analyses of different types of disorders and various aims [6,7,8,9,10,11,12,13,14,15]. For example, a recent study found that the prevalence rate of mental disorders has increased rapidly by approximately 17% in four provinces in China [15]. Although epidemiological studies on disabilities had been conducted in China, these studies collected data from parts of China. Moreover, the development of health services system was slower than economic development in China [16]. The prevalence of disabilities was aggressively increasing from 1987 to 2006. Compared with single disability, multiple disabilities would occupy more health care resource and cause more social burden. However, there is no scientific evidence of multiple disabilities that describes the prevalence and association between socioeconomic inequality and multiple disabilities risk from 1987 to 2006. In the study here, multiple disabilities was defined by the expert committee of the China National Sample Survey on Disability as more than one type of disability (Table 1), including visual, hearing and speech, physical, intellectual and mental. 

This is the first study investigating the prevalence of multiple disabilities changes over a period of twenty years and the socioeconomic inequities with multiple disabilities, using two nationally representative surveys [17,18].

## 2. Methods

### 2.1. Data Source

Till now, there were only two national representative population-based datasets from the China National Sample Surveys on Disability that were conducted in China in 1987 and 2006 [17,18]. In the present study, we used the data that were derived from these two national surveys. These surveys covered all provincial administrative areas in mainland China, excluding Hong Kong, Macau and Chinese Taipei. Both surveys used multistage, stratified random cluster sampling, with probability proportion to size to derive nationally representative samples. Within each province, sampling strata were defined based on subordinate administrative areas, local geographical characteristics or local gross domestic product, where appropriate, to allow for anticipated regional variability. Within each stratum, a four-stage sampling strategy was followed involving four natural administrative units, and sampling was conducted with probability proportional to cluster size. The sampling interval used the most up-to-date population and address information from the Ministry of Civil Affairs and Public Security in Beijing. The survey protocol and questions of this survey were reviewed by leading national and international experts, and the sampling scheme was reviewed by experts from the Division of Statistics of the United Nations [17,18]. The definition of disability in the 1987 survey was based on the International Classification of Impairment, Disability and Handicap [19], while in 2006 survey was based on the International Classification of Functioning, Disability and Health [20]. However, these two definitions are comparable [21,22]. The final sample size was 1,579,316 in the 1987 survey and 2,526,145 in the 2006 survey [17,18]. The sampling ratio of total Chinese population was 1.50 per 1000 population for the 1987 survey and 1.93 per 1000 population for the 2006 survey [17,18]. Details of the design and conduct of the studies were described elsewhere [3].

### 2.2. Ethical Statement

The surveys were approved by the State Council (Guo Ban Fa No 77 (1986) and Guo Ban Fa No 73 (2004)), conducted in all province-level administrative regions of mainland China and carried out by the Leading Group of the China National Sample Survey on Disability and the National Bureau of Statistics. The surveys were conducted within the legal framework governed by statistical law in China. All respondents provided consent to participate in the surveys. 

### 2.3. Interviewing Procedures and Data Quality

Two pilot studies were conducted in different provinces before each survey. Strict quality control measures were implemented at every step during the survey, from the drafting of the sampling frame to field sampling, from the filling out of the questionnaires to the checking of the returned forms, and from data input to the checking of data quality [17,18]. During data collection, trained field interviewers, including a team leader, a deputy team leader, a deputy medical team leader, investigators, statisticians, and medical doctors with expertise in otolaryngology, ophthalmology, surgery/orthopedics, psychiatry, and pediatrics, used a structured questionnaire to inquire about disabilities. Trained field interviewers used a structured questionnaire to inquire about visual, hearing and speech disability, physical or intellectual disability and mental disability (Table 1). Those who responded “yes” to any of the corresponding questions were assigned to different designated physicians for further disability screening and confirmation. The designated physicians performed medical examinations, made final diagnoses, assessed the severities of the disabilities (if any) following the guidelines of diagnostic manuals, and confirmed the primary causes of the disabilities Respondents with multiple positive diagnoses were examined by multiple specialists (a separate physician for each disability). In the study here, multiple disabilities was defined by the expert committee of the China National Sample Survey on Disability as more than one type of disability (Table 1), including visual, hearing and speech, physical, intellectual and mental. After the field investigations were concluded, the teams made home re-visits to conduct surveys for post-survey quality checks and calculate errors in the survey overall. The results of the quality checks showed that the omission rate of the resident population was 1.06 per 1000 persons in 1987 and 1.31 per 1000 persons in 2006; the omission rate of the disabled population was 1.16 per 1000 persons in 1987 and 1.12 per 1000 persons in 2006 [17,18].

### 2.4. Statistical Analysis

We defined the status of multiple disabilities as binary, i.e., yes or no; age group as 15–19 (yes or no), 20–24 (yes or no), 30–34 (yes or no), 35–39 (yes or no), 40–44 (yes or no), 45–49 (yes or no), 50–54 (yes or no), 55–59 (yes or no) and 60–64 (yes or no); gender as male or female; residential area as urban or rural; ethnicity as Han or others; education level as never attended school (yes or no), primary school (yes or no), junior high school and above (yes or no); marital status as never married (yes or no), divorced/widowed (yes or no) and married(yes or no); household size as 1–3 (yes or no), 4–6 (yes or no) and 7–9 (yes or no) (persons/household); living arrangement as living with others or living alone; currently employment status as binary, i.e., employed or unemployed.

The structure of the Chinese population is changing dramatically after the implementation of the one child policy. Therefore, in the present study, we examined the population aged between 15 and 64 years, because this age range influenced social production behavior, saving behavior, consumption behavior and investment behavior etc. in the two surveys. We used standard weighting procedures calculating the inverse probability of inclusion for individual survey respondent in the multistage sampling frame to construct sample weights taking into account the complex survey sample design and [23]. Standard errors were estimated by Taylor series linearization method [24]. The sample weights were adjusted by survey year when these two surveys combined. Descriptive statistics were used to present the sample characteristics, population weighted numbers by various socioeconomic characteristics. The difference proportion between the 1987 survey and 2006 survey was tested by chi-square test. Allowing for changes in the age structure of the Chinese population, we calculated the age-adjusted prevalence of multiple disabilities through direct standardization by using the 2000 China population census as the standard [25]. Logistic regression model was used to calculate the adjusted Odd Ratios (OR) and 95% Confidence Interval (CI) of each socioeconomic characteristic. The procedure SURVEYFREQ and the procedure SURVEYLOGISTIC were used to perform the data analyses. Statistical significance was declared if 2-sided *p* was <0.05. Statistical analyses were performed using SAS v. 9.2 (SAS Institute Inc., Cary, NC, USA).

## 3. Results

Selected characteristics of the population aged 15–64 years are presented in Table 2. In the study here, the crude prevalence of multiple disabilities was 4.2 per 1000 persons (95% CI: 4.0–4.3) in the 1987 survey and 6.4 per 1000 persons (95% CI: 6.2–6.5) in the 2006 survey. Age-adjusted prevalence of multiple disabilities was 4.3 per 1000 persons (95% CI: 3.9–4.7) in the 1987 survey and 6.1 per 1000 persons (95% CI: 5.6–6.6) in the 2006 survey. The age structure, educational level and household size changed significantly over the twenty years. In both surveys, male subjects, rural residents, people living with others and Han nationality accounted for the major proportion. Disabled people with two types of disabilities accounted for a major proportion of multi-disabilities in both surveys. The top three combinations of multiple disabilities, which were visual with hearing & speech, intellectual with hearing & speech and physical with hearing & speech, did not change in the twenty years. According to these two surveys’ report, around 90% of the disabled population did not access any kind of medical service or rehabilitation training in the 1987; 64% of disability population did not access medical services and 92% of the disabled population did not access rehabilitation training in 2006 (data not shown).

**Figure 1:**
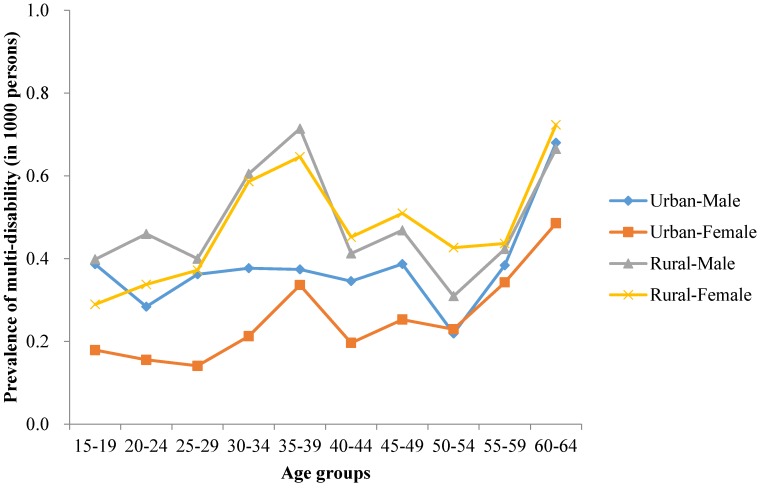
1987 age standardized prevalence of multi-disability in the Chinese population aged 15–64.

**Figure 2:**
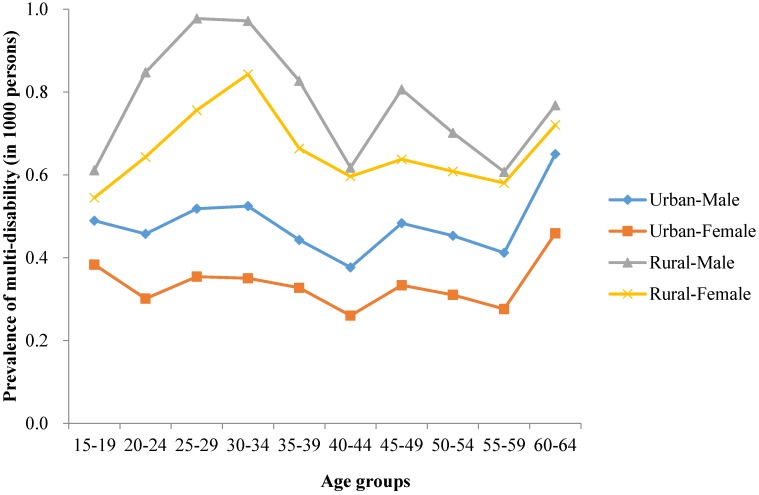
2006 age standardized prevalence of multi-disability in Chinese population aged 15–64.

A greater proportion of rural residents than urban residents and a greater proportion of males than females were affected by multiple disabilities in both surveys (Figure 1 and Figure 2). In 1987, the peak of age-adjusted prevalence was in 35–39 years. However, there were two peaks of age-adjusted prevalence in 2006. One was appeared in the age group 20–24 years and 25–29 years, the other one was in 45–49 years. In comparison with 1987, the first peak prevalence of multiple disabilities in 2006 appeared earlier.

There was a strong and consistent association between age group, gender, residence area, education and multiple disabilities with or without adjustment (Table 3 and Table 4). We observed that there was little difference in the association between age and multiple disabilities. The highest risk of multiple disabilities appeared among those aged 20–49 years in 1987 and among 25–54 years in 2006. Furthermore, male subjects were more likely to be exposed to multiple disabilities than female subjects. Residence area was also a major risk factor of multiple disabilities in the study. People with less education significantly increased the risk of multiple disabilities in both the 1987 and the 2006 survey, and the strong association remained after combining the two surveys together. Additionally, minority ethnicity increased the risk of multiple disabilities without considering other socioeconomic factors (Table 3). However, this association inversed after considering other socioeconomic factors in the 2006 survey (Table 4).

## 4. Discussion

Using detailed personal interviews and professional examinations of disabilities from the 1987 and the 2006 nationally representative sample, we obtained valuable data on multiple disabilities among the Chinese population. In the study here, the age-adjusted prevalence of multiple disabilities was lower than the average prevalence in the world [1] and increased nearly 1.5 times in twenty years. One potential reason for low prevalence is that the definition of disabilities in China is narrower than in other countries, which might lead to underestimation of the prevalence of multiple disabilities in China. Another reason is that the awareness of disabilities in China strengthened in this time period. Furthermore, the current under-development status of the health service system in China, measured as the number of hospital beds per 1000 people, the number of health facilities per 1000 people or the number of health professionals per 1000 people, is serious. For example, the number of health professionals per 1000 people only increased from 4.2 to 4.3 in twenty years [26], while during the same time period, the Chinese population increased by 20% [26]. The development of health services could not catch up with population increase. China initiated economic reforms and opened its borders to greater international trade before the 1990s. In the 1980s, the health, civil affairs and public security sectors set up a three-tier network for the prevention and treatment of psychoses. After the 1990s, for-profit hospitals were encouraged as part of the market economy during aggressive economic reforms. However, the social security system for people with disabilities and the rehabilitation system was slow to develop in China during the economic development. In 1985, the number of special education schools for disabilities was only 375 and there were only 9000 disabled students in those schools [27]. However, it was estimated that the disabled population of school age in 1985 was 6,000,000. Although the number of special education schools had increased to 1618 in 2006, it still could not meet the needs of the disabled population. This was also the reason that education inequality was significantly associated with multiple disabilities in both survey years. According to the China Disabled Persons’ Federation’s report, only 12.3% of disabled children aged 6–17 years, with access to education in 2007, were multiple disabilities children. The majority of people with disabilities never access rehabilitation services and health care even now [27], which might cause the enhancement of multiple disabilities. Moreover, the preventive disabilities services were undeveloped. Therefore, the health services system should be further improved, especially for those with multiple disabilities.

Generally, the disability prevalence has been increasing among people aged 45 and older in low- and high-income countries, especially among those aged over 55 years [1]. In the study here, the age-adjusted prevalence rate increased among those aged over 55 years as the WHO suggested in 1987. Interestingly, however, we observed a second peak in the age-adjusted prevalence rate in 2006 among those aged 45–49 years after the first peak at 20–29 years, slightly decreasing at 55–59 years and then increasing again. We also observed that the age-adjusted prevalence rate was lower in females than males across the age groups or urban-rural areas. However, according to the WHO report on disability, the prevalence of disability was higher in females than in males around the world, because females are usually more vulnerable [1]. Compared with males, the risk of multiple disabilities decreased by 13%–18% in females without considering other adjusted characteristics. This association did not change even after adjustment. Furthermore, we conducted stratified analyses with respect to education level (junior high school and above, primary school and never attended school) and currently employment status (unemployment/employment) in males and females. Gender differences in the stratified analysis still did not change. In the present study, the gender-difference on the risk of multiple disabilities might have been due to chance.

Very limited studies which examine relationships between urban/rural residence and disability in developing countries are available [28]. In our study, one striking finding was the urban-rural discrepancy in multiple disabilities prevalence over time although the urban-/rural- prevalence indicated that the contribution of urbanization to overall disability was small. For those of working age, the age-adjusted prevalence of multiple disabilities among rural residents was two times higher than that of urban residents in either 1987 or 2006. Potential explanations for the widening urban-rural gap in disability risk and prevalence in China include: (1) rural residents with multiple disabilities are unable to migrate to urban areas; and (2) harder lifestyle, poorer working standards, as well as limited availability of healthcare and rehabilitation services in rural areas [29]. The discrepancy observed in the present study is consistent with the results of the Fourth National Health Services Survey in 2008, which found higher rates of disabled people among rural residents [30]. Although the health services system has been gradually developing, it still cannot catch up with residents’ health care demands and utilization, especially for those living with disabilities in rural areas. Our results suggest that governments should improve health services system in rural areas.

People with disabilities usually have poorer health conditions/outcomes, lower educational level, less economic participation and higher risk of poverty than those without disabilities [1]. A possible explanation for these outcomes is that people with disabilities experience barriers in accessing services such as health, education, employment, and so on [1]. Furthermore, education inequalities are also associated with disabilities. An epidemiological study suggested that higher education provided protection against developing disability, but provided less benefit when disability was already present, especially for aged people [31]. The study also described a high incidence of disabilities among those with lower educational attainment (i.e., less than secondary education) in the population. Other studies have suggested a gradient in disability by education, occupation, and material living standards [32,33].

In the present study, we observed results consistent with previous epidemiological studies when the surveys were analyzed both separately and together. People with single or multiple disabilities have diverse personal factors with differences in socioeconomic status or ethnicity [1]. In the present study, minority ethnicity was identified as a possible risk factor for multiple disabilities, although this association was not substantial. In China, compared with people of the Han ethnicity, those of minority ethnicities, especially those with disabilities, are more likely to live in poverty with less access to social security and education [34]. Socioeconomic inequalities were not more serious in the 1987 than in the 2006. Therefore, we observed minority ethnicity was only a risk factor of multiple disabilities in the 1987 survey. Our results suggested that socioeconomic factors, such as education level residential area employment status, might contribute to multiple disabilities more than ethnicity did.

## 5. Limitations

This study has provided a broad understanding of the prevalence multiple disabilities and its relationship with different key socioeconomic factors in China in a twenty-year period. Moreover, the current study used a large, representative population-based sample covering all the provincial areas. In addition, every subject of the selected households was interviewed by interviewers face to face. Subjects who agreed to participate in the study underwent a screening of disabilities by interviewers, and those suspected to have a disability were then examined and diagnosed by doctors. However, the present study also had some weaknesses, such as the use of the International classification of impairments, disabilities, and handicaps in the 1987 survey [19] and the international classification of functioning, disability and health in the 2006 survey [20] to classify disability. However, both surveys consistently employed the Chinese word “Canji”, which means both handicap and disability, in the definitions used in both surveys. These differences should be taken into consideration for further studies. The more stringent definition of disability used in both surveys caused a low prevalence of multiple disabilities compared with other researches on disability, which should serve to inform future studies. Moreover, standardized quality control schemes were in place during the field implementation such as training of the interviewers and crosscheck the returned survey responses by contacting survey participants, resulting in little response bias. Additionally, the cross-sectional design does not provide direct evidence of causality.

## 6. Conclusions

Currently, China is undergoing social reforms; these results will be beneficial for understanding trends in multiple disabilities and socioeconomic indicators over a twenty-year period in China. The widening gap in prevalence rates of multiple disabilities between rural and urban areas may have important practical implications for China. In addition, the study results may help the government adjust strategies aimed at assisting individuals and communities and improving health care systems to prevent multi-disabilities and/or improve the lives of the disabled population, especially for rural residents.

## Figures and Tables

**Table 1:** Study definitions for different disability types and their corresponding survey questions [3].

Disability Type	Survey Question	Definition
Visual	Do you or your family members have visual problems?	Visual disability refers to poor vision or constriction of the visual field in both eyes from any cause and is not correctable. It consists of two categories: Blindness and weak vision.
Hearing and speech	Do you or your family members have hearing or speech problems?	Hearing disability refers to permanent hearing loss of varying degrees from any cause or the inability to hear at all or to hear clearly any nearby sound or voice. These deficits affect daily life and social activities. Speech disability refers to any type of language disorder. Because successful treatment takes more than 1 year and the disability is generally present for more than 2 years, the patient cannot take part in normal language exchanges, which undermines his or her daily life and participation in social activities.
Physical	Do you or your family members have any difficulty walking, standing, squatting, climbing the stairs, grasping, washing and rinsing, or dressing?	Physical disability refers to a loss of motor function of varying degrees or to limitations in movements or activities resulting from deformed limbs or body paralysis (palsy) or from deformity caused by damage to the structure or function of those body parts involved in mobility.
Intellectual	Do you or your family members have any difficulty studying?	Intellectual disability refers to lower than normal intellectual ability and is accompanied by adaptive behavior disorders. This kind of disability results from impairment of the structure and functions of the nervous system, limits individual activity and participation, and requires all-round, extensive, limited, or intermittent support.
Mental	Are you or your family members forgetful? Or do you have difficulty concentrating? Or can you not control your moods? Or do you have strange behavior that is out of the ordinary? Or are you addicted to alcohol or drugs?	Mental disability refers to psychiatric disorders lasting more than 1 year that manifest as cognitive, affective, and behavior disorders affecting the daily life and social participation of the patient.

**Table 2:** Characteristics of the weighted national survey population ^a^.

Age Groups (Years)	1987	2006
	Weighted Sample Size, *n* (%)	Weighted Sample Size, *n* (%)
60–64	30,159,817 (4.5)	54,198,921 (5.8)
55–59	37,727,881 (5.6)	73,812,498 (8.0)
50–54	43,974,140 (6.6)	99,313,597 (10.7)
45–49	45,323,215 (6.8)	86,458,080 (9.3)
40–44	50,246,799 (7.5)	117,955,746 (12.7)
35–39	67,428,705 (10.1)	124,648,044 (13.4)
30–34	86,717,556 (12.9)	106,183,740 (11.4)
25–29	69,689,449 (10.4)	79,950,588 (8.6)
20–24	115,229,729 (17.2)	74,541,801 (8.0)
15–19	124,302,057 (18.5)	110,314,115 (11.9)
*p* (1987 vs. 2006)	<0.01	
Gender		
Male	337,122,104 (50.3)	466,563,760 (50.3)
Female	333,677,243 (49.7)	460,813,370 (49.7)
*p* (1987 vs. 2006)	0.39	
Residence, %		
Urban	202,323,830 (30.2)	297,549,946 (32.1)
Rural	468,475,517 (69.8)	629,827,185 (67.9)
*p* (1987 vs. 2006)	0.07	
Ethnicity, %		
Han	616,920,610 (92.0)	839,420,958 (90.5)
Others	53,878,737 (8.0)	87,956,172 (9.5)
*p* (1987 vs. 2006)	0.003	
Education, %		
Junior high school and above	269,298,712 (40.1)	581,462,856 (62.7)
Primary school	218,900,503 (32.6)	254,587,511 (27.5)
Never attended school	182,600,132 (27.2)	91,326,764 (9.8)
*p* (1987 vs. 2006)	<0.01	
Marital status, %		
Married	45,734,448 (68.1)	709,249,061 (76.5)
Divorced or widowed	22,545,344 (3.4)	32,425,867 (3.5)
Never married	190,909,555 (28.5)	185,702,203 (20.0)
*p* (1987 vs. 2006)	<0.01	
Household size		
1–3	141,391,883 (21.1)	422,825,807 (45.6)
4–6	404,126,746 (60.2)	464,977,622 (50.1)
7–9	125,280,718 (18.7)	39,573,702 (4.3)
*p* (1987 vs. 2006)	<0.01	
Living arrangement		
Living with others	664,348,720 (99.0)	908,678,415 (98.0)
Living alone	6,450,627 (1.0)	18,698,715 (2.0)
*p* (1987 vs. 2006)	<0.01	
Currently employed, %		
Yes	534,142,450 (79.6)	710,108,470 (76.6)
No	136,656,897 (20.4)	217,268,660 (23.4)
*p* (1987 vs. 2006)	<0.01	

^a^ weighted national survey population included multiple disabilities population and without any type of disabilities’ population.

**Table 3:** Crude odds ratios of multiple disabilities (yes/no) according to the characteristics in the study.

Characteristics of the Survey Population	Reference	OR (95% CI), 1987	OR (95% CI), 2006
Age groups (years)			
60–64	No	**3.70 (3.37–4.05)**	**2.40 (2.26–2.55)**
55–59	No	**1.92 (1.73–2.12)**	**1.57 (1.48–1.68)**
50–54	No	1.05 (0.93–1.19)	**1.23 (1.16–1.31)**
45–49	No	1.07 (0.94–1.21)	0.96 (0.90–1.03)
40–44	No	0.98 (0.87–1.11)	**0.83 (0.77–0.88)**
35–39	No	**1.14 (1.02–1.29)**	**0.74 (0.69–0.79)**
30–34	No	**0.80 (0.71–0.89)**	**0.76 (0.71–0.82)**
25–29	No	**0.58 (0.51–0.66)**	**0.81 (0.75–0.88)**
20–24	No	**0.74 (0.66–0.82)**	0.94 (0.87–1.01)
15–19	No	**0.61 (0.55–0.68)**	**0.69 (0.64–0.75)**
Gender	Male	**0.87 (0.81–0.92)**	**0.81 (0.78–0.85)**
Residence	Urban	**1.47 (1.34–1.62)**	**1.74 (1.63–1.85)**
Ethnicity	Han	**1.27 (1.09–1.49)**	**1.32 (1.22–1.43)**
Education			
Junior high school and above	No	**0.07 (0.06–0.08)**	**0.10 (0.09–0.11)**
Primary school	No	**0.30 (0.27–0.34)**	**0.83 (0.79–0.87)**
Never attended school	No	**12.75 (11.66–13.95)**	**14.90 (14.22–15.61)**
Marital status			
Married	No	**0.39 (0.36–0.41)**	**0.31 (0.30–0.32)**
Divorced or widowed	No	**3.15 (2.83–3.51)**	**2.25 (2.09–2.42)**
Never married	No	**2.05 (1.91–2.20)**	**2.93 (2.81–3.06)**
Household size			
1–3	No	**1.48 (1.37–1.59)**	**1.11 (1.06–1.16)**
4–6	No	**0.77 (0.73–0.83)**	**0.87 (0.83–0.90)**
7–9	No	0.93 (0.86–1.02)	**1.26 (1.14–1.38)**
Living arrangement	Living with others	**4.09 (3.43–4.87)**	**2.81 (2.56–3.08)**
Currently employed	Yes	**9.11 (0.43–9.84)**	**6.21 (5.94–6.50)**

Abbreviations: CI = confidence interval; OR = odds ratio; Bold writing: *p* < 0.05.

**Table 4:** Associations of multiple disabilities (yes/no) in Chinese population aged 15–64.

1987 and 2006 Year	Reference		OR (95% CI)
Investigation year	1987	2006	**2.96 (2.79–3.14)**
Age group (years)	60–64	55–59	0.98 (0.92–1.06)
		50–54	1.06 (0.98–1.14)
		45–49	**1.44 (1.33–1.56)**
		40–44	**1.96 (1.80–2.14)**
		35–39	**1.80 (1.64–1.96)**
		30–34	**1.69 (1.54–1.85)**
		25–29	**1.56 (1.41–1.73)**
		20–24	**1.16 (1.03–1.30)**
		15–19	**0.37 (0.32–0.41)**
Gender	Male	Female	**0.44 (0.42–0.46)**
Residence	Urban	Rural	**1.15 (1.09–1.22)**
Ethnicity	Han	Others	1.08 (1.00–1.16)
Education	High school and above	Primary school	**6.94 (6.43–7.51)**
		Never attended school	**45.67 (41.89–49.79)**
Marital status	Married	Divorced or widowed	**1.49 (1.39–1.59)**
		Never married	**6.72 (6.27–7.19)**
Household size	1–3	4–6	**0.89 (0.85–0.92)**
		7–9	**0.85 (0.78–0.91)**
Living arrangement	Living with others	Living alone	**0.62 (0.56–0.69)**
Currently employed	Yes	No	**8.53 (8.13–8.95)**
**1987**			
Age group (years)	60–64	55–59	**0.87 (0.76–0.99)**
		50–54	**0.79 (0.67–0.92)**
		45–49	**1.48 (1.27–1.73)**
		40–44	**2.06 (1.75–2.42)**
		35–39	**2.57 (2.18–3.03)**
		30–34	**2.00 (1.70–2.36)**
		25–29	**1.67 (1.39–2.01)**
		20–24	**1.28 (1.04–1.56)**
		15–19	**0.29 (0.23–0.36)**
Gender	Male	Female	**0.27 (0.25–0.30)**
Residence	Urban	Rural	1.08 (0.98–1.20)
Ethnicity	Han	Others	**1.29 (1.10–1.51)**
Education	High school and above	Primary school	**7.94 (6.60–9.54)**
		Never attended school	**79.49 (64.57–97.86)**
Marital status	Married	Divorced or widowed	**1.72 (1.52–1.96)**
		Never married	**15.72 (13.83–17.87)**
Household size	1–3	4–6	**0.77 (0.71–0.84)**
		7–9	**0.69 (0.62–0.78)**
Living arrangement	Living with others	Living alone	**0.46 (0.37–0.57)**
Currently employed	Yes	No	**18.88 (16.99–20.99)**
**2006**			
Age group (years)	60–64	55–59	1.08 (0.99–1.17)
		50–54	**1.23 (1.13–1.34)**
		45–49	**1.57 (1.42–1.73)**
		40–44	**2.12 (1.92–2.34)**
		35–39	**1.72 (1.56–1.90)**
		30–34	**1.80 (1.62–2.01)**
		25–29	**1.76 (1.56–1.99)**
		20–24	**1.16 (1.02–1.33)**
		15–19	**0.41 (0.35–0.47)**
Gender	Male	Female	**0.50 (0.48–0.53)**
Residence	Urban	Rural	**1.19 (1.11–1.23)**
Ethnicity	Han	Others	1.01 (0.94–1.10)
Education	High school and above	Primary school	**6.47 (5.93–7.07)**
		Never attended school	**39.70 (36.12–43.63)**
Marital status	Married	Divorced or widowed	**1.39 (1.28–1.51)**
		Never married	**5.14 (4.75–5.56)**
Household size	1–3	4–6	**0.95 (0.90–0.99)**
		7–9	0.97 (0.87–1.08)
Living arrangement	Living with others	Living alone	**0.70 (0.62–0.78)**
Currently employed	Yes	No	**7.44 (7.02–7.88)**

Abbreviations: CI = confidence interval; OR = odds ratio; Bold font: *p* < 0.05.
